# Supplementary material for: Modelling upper respiratory viral load dynamics of SARS-CoV-2
Source: BMC Med. 2022 Jan 13;20:25. doi: 10.1186/s12916-021-02220-0 (PMC8755404; doi:10.1186/s12916-021-02220-0)
Supplement: Supplementary file 1 — Additional file 1: Supplementary Table S1. Summary of studies which were identified during the literature search but did not provide viral load data when the corresponding authors were contacted. Studies included both male and female patients, unless stated. All ages stated are in years. *For some studies, particularly those carried out early in the pandemic, patients were hospitalised to ensure isolation (i.e. cohorts may include asymptomatic subjects or those with very mild symptoms). † These studies were identified during our literature search, but we were informed by the study authors that quantitative viral load was not available (i.e. only positive or negative result recorded). Supplementary Table S2. Summary of antiviral and immunomodulatory treatment in the studies included in analysis. Supplementary Table S3. Summary of the population-level (i.e. not study- or patient-specific) parameter values (and 95% credible intervals) obtained for the multi-level regression modelling (as displayed in Fig. 2). Patient- and study-specific random effects were used for both the peak (log-transformed) viral load, and its rate of decline per day. Supplementary Table S4. assessing the goodness of fit of the regression models using leave-one-out cross-validation. Supplementary Table S5. Summary of the population-level (i.e. not study- or patient-specific) parameter values (and 95% credible intervals) obtained for the mechanistic viral load model (Eqs. 6–8). Samples from ${k}_a^0$ka0 and [file 12916_2021_2220_MOESM1_ESM.pdf]

<u>**Modelling upper respiratory viral load dynamics of SARS-CoV-2**</u>

Joseph D. Challenger[1*], Cher Y. Foo[2], Yue Wu[3], Ada W. C. Yan[4], Mahdi Moradi Marjaneh[5], Felicity Liew[6], Ryan S. Thwaites[6], Lucy C. Okell[1], Aubrey J. Cunnington[5,7]

1. Medical Research Council Centre for Global Infectious Disease Analysis, Department of Infectious Disease Epidemiology, Imperial College London, London, United Kingdom

2. School of Medicine, Imperial College London, London, United Kingdom

3. School of Clinical Medicine, University of Cambridge, Cambridge, United Kingdom

4. Department of Infectious Disease, Imperial College London, London, United Kingdom

5. Section of Paediatric Infectious Disease, Department of Infectious Disease, Imperial College London, London, United Kingdom

6. National Heart and Lung Institute, Imperial College London, United Kingdom

7. Centre for Paediatrics and Child Health, Imperial College London, United Kingdom

**Corresponding author*

SUPPLEMENTARY MATERIALS

This document contains:

1. Supplementary Tables 1-5
2. Supplementary Figures 1-8

All references numbered as per the main text

Title	Journal	D.O.I.	No. of subjects	Study population	Study Setting	Date of request
Temporal dynamics in viral shedding and transmissibility of COVID-19	Nature Medicine	https://doi.org/10.1038/s41591-020-0869-5	94	Inpatients, age range unclear (median age=47)	Guangzhou, China	May 26th 2020
Temporal profiles of viral load in posterior oropharyngeal saliva samples and serum antibody responses during infection by SARS-CoV-2: an observational cohort study	Lancet ID	https://doi.org/10.1016/S1473-3099(20)30196-1	23	Inpatients, age range 37-75 (median=62)	Hong Kong	Oct 22nd 2020
Prolonged presence of SARS-CoV-2 viral RNA in faecal samples	Lancet G Hep	https://doi.org/10.1016/S2468-1253(20)30083-2	98	Inpatients, age range unclear (median=44)	Zhuhai, China	June 1st 2020
Clinical Course and Outcomes of Patients with Severe Acute Respiratory Syndrome Coronavirus 2 Infection: a Preliminary Report of the First 28 Patients from the Korean Cohort Study on COVID-19	J Korean Med Sci	https://doi.org/10.3346/jkms.2020.35.e142	28	Inpatients, age range 20-73 (median=40)	Korea (nationwide)	June 1st 2020
Viral Kinetics and Antibody Responses in Patients with COVID-19	medRxiv	https://doi.org/10.1101/2020.03.24.20042382	67	Inpatients, age range 10-77 (median=49)	Chongqing, China	May 26th 2020
Different longitudinal patterns of nucleic acid and serology testing results based on disease severity of COVID-19 patients	Emerging Microbes & Infections	https://doi.org/10.1080/22221751.2020.1756699	21†	Inpatients, age range 10-73 (median=37)		June 1st 2020
The presence of SARS-CoV-2 RNA in the feces of COVID-19 patients	Journal of Medical Virology	https://doi.org/10.1002/jmv.25825	42	Inpatients, age range unclear (median=51)	Wuhan, China	Oct 21st 2020
Chronological Changes of Viral Shedding in Adult Inpatients with COVID- 19 in Wuhan, China	Clinical Infectious Diseases	https://doi.org/10.1093/cid/ciaa631	308	Adult inpatients, age range unclear (median=58.5)	Wuhan, China	June 1st 2020
Correlation between viral RNA shedding and serum antibodies in COVID-19 patients	Clinical Microbiology and Infection	https://doi.org/10.1016/j.cmi.2020.05.022	89	Inpatients, age range unclear (median=62)	Wuhan, China	June 1st 2020
Viral load dynamics and disease severity in patients infected with SARS-CoV-2 in Zhejiang province, China, January-March 2020: retrospective cohort study	BMJ	https://doi.org/10.1136/bmj.m1443	96	Inpatients, age range unclear (median=55)	Zheijang, China	June 1st 2020
Clinical features and dynamics of viral load in imported and non-imported patients with COVID-19	International Journal of Infectious Diseases	https://doi.org/10.1016/j.ijid.2020.03.022	51	Inpatients*, age range unclear	Changzhou, China	June 1st 2020

Antibody responses to SARS-CoV-2 in patients of novel coronavirus disease 2019	Clinical Infectious Diseases	https://doi.org/10.1093/cid/ciaa344	173	Inpatients, age range unclear (median=48)	Shenzhen, China	August 20th 2020
Phenotype of SARS-CoV-2-specific T-cells in COVID-19 patients with acute respiratory distress syndrome	medRxiv	https://doi.org/10.1101/2020.04.11.20062349	10	10 inpatients (moderate-severe ARDS), age range 49-72	Location of study unclear	June 16th 2020
Shedding of infectious virus in hospitalized patients with coronavirus disease-2019 (COVID-19): duration and key determinants	medRxiv	https://doi.org/10.1101/2020.06.08.20125310	129	Inpatients, age range unclear (median=65)	Rotterdam, The Netherlands	June 16th 2020
SARS-CoV-2 viral load predicts COVID-19 mortality	Lancet Resp Med	https://doi.org/10.1016/S2213-2600(20)30354-4	1145	Inpatients, age range unclear (mean=65)	New York, United States of America	August 14th 2020
Duration of viral shedding in asymptomatic or mild cases of novel coronavirus disease 2019 (COVID-19) from a cruise ship: A single-hospital experience in Tokyo, Japan	International Journal of Infectious Diseases	https://doi.org/10.1016/j.ijid.2020.06.020	23	Inpatients*, age range 29-79, median=67	Yokohama, Japan (cohort from a cruise ship)	August 20th 2020
Relative COVID-19 viral persistence and antibody kinetics	MedRxiv	https://doi.org/10.1101/2020.07.01.20143917	15	Inpatients*, age range unclear (mean=49)	Taoyuan, Taiwan	August 19th 2020
Kinetics of viral load and antibody response in relation to COVID-19 severity	The Journal of Clinical Investigation	https://doi.org/10.1172/JCI138759	23	Inpatients, age range 24-82	Guangzhou, Yangjiang & Qingyuan (China)	August 20th
SARS-CoV-2 RT-PCR profile in 298 Indian COVID-19 patients: a retrospective observational study	MedRxiv	https://doi.org/10.1101/2020.06.19.20135905	298 (164 of whom were symptomatic)	Inpatients*, age range 0.5-88 (mean=39)	New Delhi, India	August 19th 2020
Viral load dynamics in transmissible symptomatic patients with COVID-19	MedRxiv	https://doi.org/10.1101/2020.06.02.20120014	28	Inpatients*, age range unclear (median=45)	Toyama, Japan	August 19th 2020
Clinical Course and Molecular Viral Shedding Among Asymptomatic and Symptomatic Patients With SARS-CoV-2 Infection in a Community Treatment Center in the Republic of Korea	JAMA	https://doi.org/10.1001/jamainternmed.2020.3862	303	Symptomatic & asymptomatic subjects, isolating at a community treatment centre. Age range unclear (median=25)	Cheonan, Republic of Korea	August 19th 2020
Duration of infectiousness and correlation with RT-PCR cycle threshold values in	Eurosurvellance	https://doi.org/10.2807/1560-7917.ES.2020.25.32.2001483	253	Symptomatic & asymptomatic subjects,	England (nationwide)	August 19th 2020

cases of COVID-19, England, January to May 2020				samples collected from outbreak investigations, healthcare worker surveillance and community surveillance		
Clinical Characteristics and Viral RNA Detection in Children With Coronavirus Disease 2019 in the Republic of Korea	JAMA Pediatr	https://doi.org/10.1001/jamapediatrics.2020.3988	91	Symptomatic & asymptomatic children, samples collected from contact tracing, border surveillance, and testing due to covid-19-like symptoms	Korea (nationwide)	Sept 4th 2020
Temporal profile and determinants of viral shedding and of viral clearance confirmation on nasopharyngeal swabs from SARS-CoV-2-positive subjects: a population-based prospective cohort study in Reggio Emilia, Italy	BMJ Open	http://dx.doi.org/10.1136/bmjopen-2020-040380	1162†	Samples acquired from health service database (recording both inpatient and outpatient test results)	Reggio-Emilia, Italy	Sept 9th 2020
Viral Dynamics and Immune Correlates of Coronavirus Disease 2019 (COVID-19) Severity	Clinical Infectious Diseases	https://doi.org/10.1093/cid/ciaa1280	100	Inpatients, age range unclear (median=44)	Singapore	Oct 16th 2020
Viral dynamics in mild and severe cases of COVID-19	Lancet ID	https://doi.org/10.1016/S1473-3099(20)30232-2	76	Inpatients, age range unclear (mean=48)	Nanching, China	Oct 22nd 2020
Clinical and epidemiological features of COVID-19 family clusters in Beijing, China	Journal of Infection	https://doi.org/10.1016/j.jinf.2020.04.018	24	Four family clusters identified via contact tracing, age range 0.75-86	Beijing, China	Oct 22nd 2020
Clinical and epidemiological features of 36 children with coronavirus disease 2019 (COVID-19) in Zhejiang, China: an observational cohort study	Lancet ID	https://doi.org/10.1016/S1473-3099(20)30198-5	36	Paediatric inpatients, (mean age=8.3)	Zhejiang, China	Oct 22nd 2020
Viral loads in throat and anal swabs in children infected with SARS-CoV-2	Emerging Microbes & Infections	https://doi.org/10.1080/22221751.2020.1771219	217	Paediatric inpatients, age range unclear	Wuhan, China	Oct 22nd 2020
Factors of Severity in Patients with COVID-19: Cytokine/Chemokine Concentrations, Viral Load, and Antibody Responses	AJTMH	https://doi.org/10.4269/ajtmh.20-1110	31	Inpatients, age range unclear (mean=50)	Republic of Korea	Nov 26th 2020
The kinetics of viral load and antibodies to SARS-CoV-2	Clinical Microbiology and Infection	https://doi.org/10.1016/j.cmi.2020.08.043	35	Inpatients, age and sex unclear	Guangdong, China	Nov 26th 2020

The Correlation Between Clinical Features and Viral RNA Shedding in Outpatients With COVID-19	Open Forum Infectious Diseases	https://doi.org/10.1093/ofid/ofaa331	918	Outpatients, age range unknown (mean=56)	Wuhan, China	Nov 26th 2020
Molecular and serological characterization of SARS-CoV-2 infection among COVID-19 patients	Virology	https://doi.org/10.1016/j.virol.2020.09.008	100	Inpatients, age range unknown (mean=51)	Guangzhou, China	Nov 26th 2020

Supplementary Table 1: Summary of studies which were identified during the literature search but did not provide viral load data when the corresponding authors were contacted. Studies included both male and female patients, unless stated. All ages stated are in years. *For some studies, particularly those carried out early in the pandemic, patients were hospitalised to ensure isolation (i.e. cohorts may include asymptomatic subjects or those with very mild symptoms). † These studies were identified during our literature search, but we were informed by the study authors that quantitative viral load was not available (i.e. only positive or negative result recorded).

Study	Reference	No. of Patients	Country	Healthcare setting	Recruitment period	Antiviral treatment, n (%)		Immunomodulatory treatment, n(%)	
1	Kim JY *et al.*, J Korean Med Sci, 2020 [44]	2	Korea	Hospital	January 2020	Lopinavir/Ritonavir	2 (100)	0	0
2	Lui G *et al.*, J Infect, 2020 [55]	11	Hong Kong	Multicentre, Hospital	February 2020	Lopinavir/Ritonavir<br>Ribavirin<br>Beta-interferon	11 (100)<br>8 (73)<br>5 (56)	Hydrocortisone	1 (9)
3	Scott S *et al.*, Clin Infect Dis, 2020 [56]	1	USA	Community	January 2020	None used		None used	
4	Kim SE, Int J Infect Dis, 2020 [57]	3	Korea	Tertiary Hospital	February – April 2020	Lopinavir/Ritonavir	1 (33)	None used	
5	Gautret P *et al.*, Int J Antimicrob Agents, 2020 [58]	19	France	Tertiary Hospital	March 2020	None used		Hydroxychloroquine	18 (95)
6	Young B *et al.*, JAMA, 2020 [59]	18	Singapore	Multicentre, Tertiary Hospital	January – February 2020	Lopinavir/Ritonavir	5 (28)	None used	
7	The COVID–19 Investigation team, Nat Med, 2020 [60]	12	USA	Multicentre, Community and Hospital	January 2020	Remdesivir	3 (25)	Corticosteroids	2 (17)
8	Wölfel R *et al.*, Nature, 2020 [61]	9	Germany	Hospital	January 2020	Not reported		Not reported	
9	Vetter P *et al.*, mSphere, 2020 [62]	5	Switzerland	Hospital	February 2020	Lopinavir/Ritonavir	1 (20)	None used	
10	Lavezzo E *et al.*, Nature, 2020 [45]	37	Italy	Community and Hospital	February – March 2020	Not reported		Not reported	
11	Xu Y *et al.*, Nat Med, 2020 [63]	6	China	Paediatric cohort, Tertiary Hospital	January – February 2020	Alpha-interferon	6 (100)	IVIG	1 (17)
12	Shrestha N *et al.*, Clin Infect Dis, 2020 [64]	230	USA	Healthcare worker cohort, non–hospitalized	March – April 2020	None used		None used	
13	Fajnzylber J *et al.*, Nat Commun, 2020 [65]	64	USA	Multicentre, Tertiary Hospital	NK	Remdesivir	16 (25)	None used	
14	Yilmaz A et *al.*, J Infec Dis, 2020 [66]	54	Sweden	Tertiary Hospital	February – April 2020	Not reported		Not reported	
15	Alsharrah *et al.*, J Med Virol, 2020 [67]	29	Kuwait	Paediatric cohort, Tertiary Hospital	February – April 2020	None used		None used	
16	Tan A *et al.*, Cell Reports, 2020 [7]	12	Singapore	Tertiary Hospital	NK	Not reported		Not reported	
17	Salvatore PP *et al.*, Clin Infec Dis, 2020 [68]	93	USA	Community	March – May 2020	None used		None used	

Supplementary Table 2. Summary of antiviral and immunomodulatory treatment in the studies included in analysis.

Parameter	Interpretation	Posterior Mean	95% CrI
a_0	Average value of the peak $\log_{10}$(VL)	6.74	(6.17,7.30)
b_0	Average value of the rate of decline (per day) of the $\log_{10}$(VL)	-0.22	(-0.26,-0.17)
$\sigma_{patient}[1]$	Standard deviation of the between-patient variation in the peak $\log_{10}$(VL)	1.54	(1.26,1.85)
$\sigma_{patient}[2]$	Standard deviation of the between-patient variation rate of decline (per day) of the $\log_{10}$(VL)	0.15	(0.12,0.19)
$\rho_{patient}$	Correlation between $\sigma_{patient}[1]$ and $\sigma_{patient}[2]$	-0.86	(-0.92,-0.76)
$\sigma_{study}[1]$	Standard deviation of the between-study variation in the peak $\log_{10}$(VL)	0.81	(0.42,1.35)
$\sigma_{study}[2]$	Standard deviation of the between-study variation rate of decline (per day) of the $\log_{10}$(VL)	0.04	(0.01,0.09)
ρ_{study}	Correlation between $\sigma_{study}[1]$ and $\sigma_{study}[2]$	-0.18	(-0.80,0.45)
σ	Standard deviation of the observed variation in $\log_{10}$(VL) around the linear model	1.08	(1.01,1.16)

Supplementary Table 3: Summary of the population-level (i.e. not study- or patient-specific) parameter values (and 95% credible intervals) obtained for the multi-level regression modelling (as displayed in Figure 2). Patient- and study-specific random effects were used for both the peak (log-transformed) viral load, and its rate of decline per day.

Data from all studies (n=16)		
Fixed Effects Included	$\Delta ELPD_{loo}$	dSE
None	-	-
Severity	-0.8	0.7
**Data from studies (n=14) with demographic information**		
Fixed Effects Included	$\Delta ELPD_{loo}$	dSE
None	-	-
Age	-2.1	0.6
Sex	-2.4	0.8
Severity	-2.5	0.8
Severity + Age	-3.2	0.9
Severity + Sex	-3.5	0.9
Age + Sex	-3.6	0.7
Age + Severity + Sex	-4.5	1.1

Supplementary Table 4: assessing the goodness of fit of the regression models using leave-one-out cross-validation. In Table 2, we show the goodness of fit according to the Watanabe Akaike Information Criterion (WAIC). Here, we compare those results with results obtained by leave one out cross-validation [69]. As before, we carried out two separate model comparisons, as only 14 of the 16 studies provided individual-level demographic data. The analysis was carried out using the 'loo' package in R, using the PSIS-LOO approximation to exact LOO-CV. The expected log predictive density ($ELPD_{loo}$) is calculated: the model that provides the best fit to the data has the highest value. Here we show the difference measured in the goodness of fit between the best model and all the others ($\Delta ELPD_{loo}$). We also show the standard error of this difference (dSE). As per the WAIC analysis, we found that the inclusion of fixed effects for age, sex, or severity (either separately or in linear combination) did not improve the model fit.

Parameter	Interpretation	Posterior Mean	95% CrI
k_a^0	Population-level late immune response	6.32	(5.84-6.74)
σ_{kp}	Standard deviation of patient-level offset in the late immune response	13.62	(12.40-15.02)
σ_{ks}	Standard deviation of study-level offset in the late immune response	0.74	(0.49-1.04)
I_{max}^0	Population-level late immune response	0.66	(0.31-1.12)
σ_{Ip}	Standard deviation of patient-level offset in the early immune response	2.81	(2.37-3.26)
σ_{Is}	Standard deviation of study-level offset in the early immune response	2.85	(1.92-4.10)
σ	Standard deviation of variation of viral load around modelled trajectory	3.21	(3.00-3.43)

Supplementary Table 5: Summary of the population-level (i.e. not study- or patient-specific) parameter values (and 95% credible intervals) obtained for the mechanistic viral load model (Equations 6-8). Samples from k_a^0 and I_{max}^0 were used to generate the black line and dark grey shaded area in Figure 4.

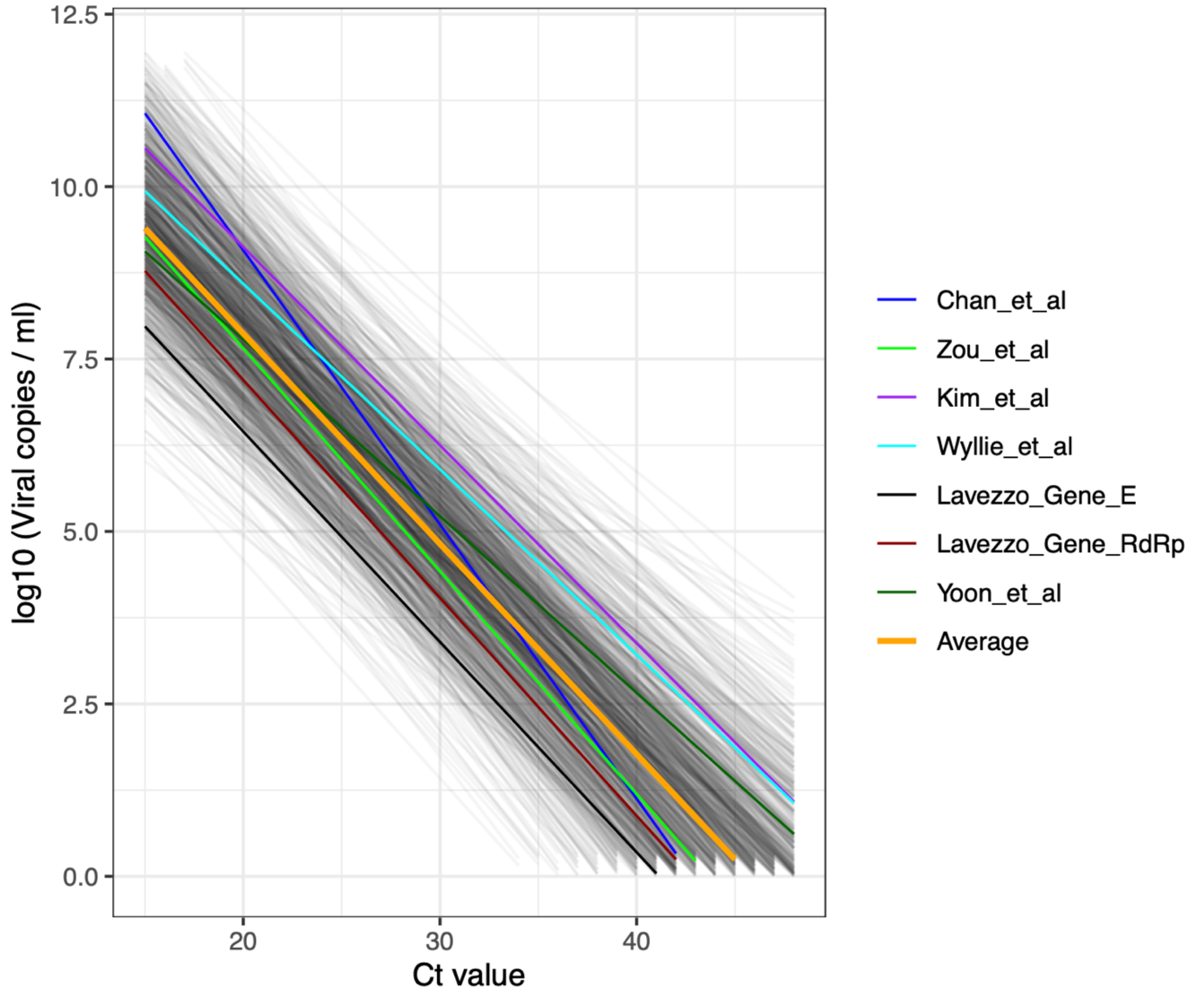

Supplementary Figure 1: Standard curves relating cycle-threshold (Ct) values to viral load. Seven standard curves, identified from published studies (see Methods) are plotted. We used these standard curves to produce a model of an averaged standard curve (thicker orange line), and capture the variation observed across different studies. We only used standard curves which quantified viral load in units of viral copies per ml. Standard curves generated by drawing random samples from this model are shown by opaque, grey lines, indicating the potential variation in the standard curve.

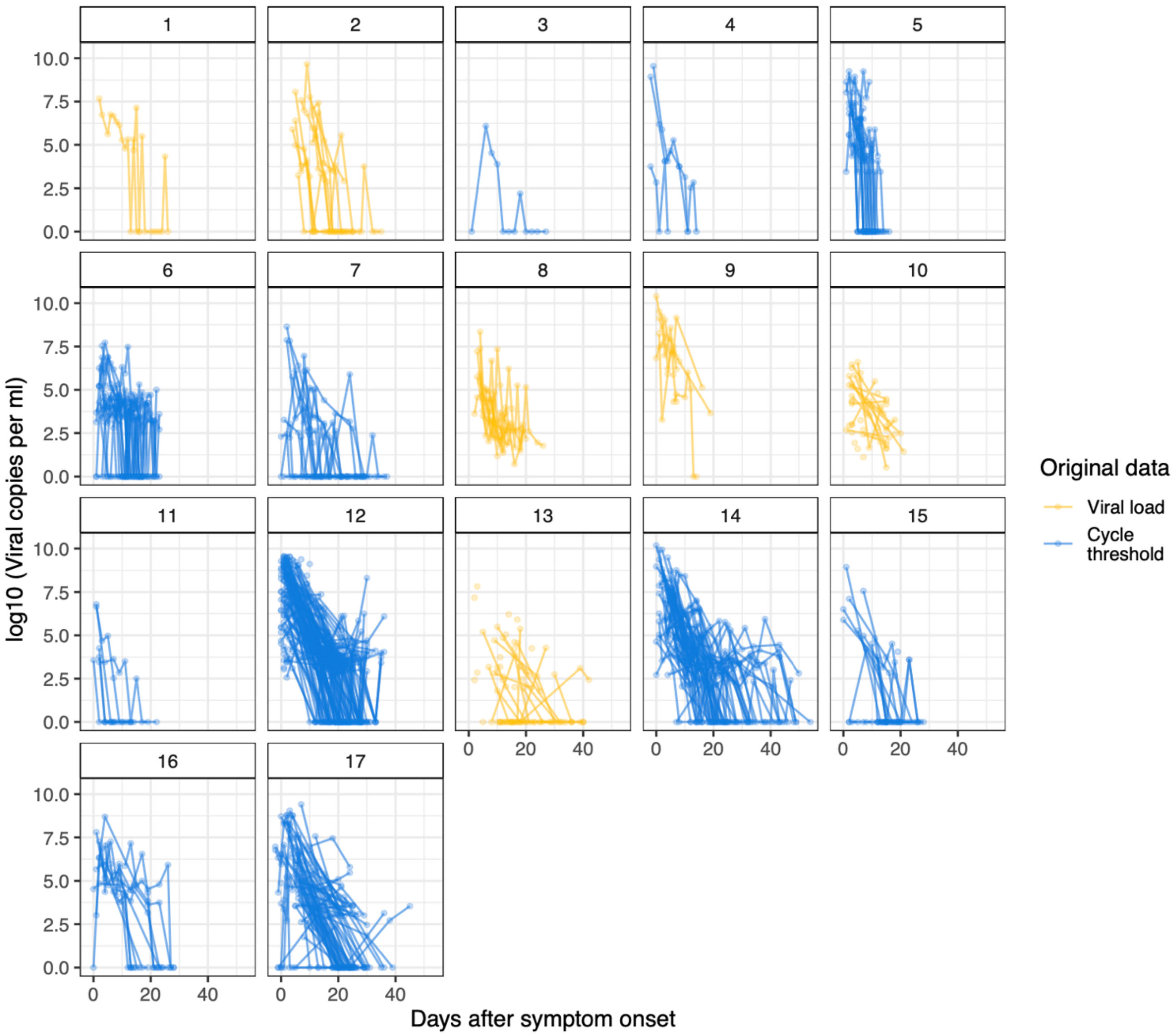

Supplementary Figure 2: Summary of all the data collected (see Table 1 in the main text). For the studies shown in blue, viral loads have been estimated using an averaged standard curve (see Methods for details). For illustrative purposes, samples that were negative for virus are set to 1 viral copy per ml. In each panel, the lines connect samples collected from the same patient.

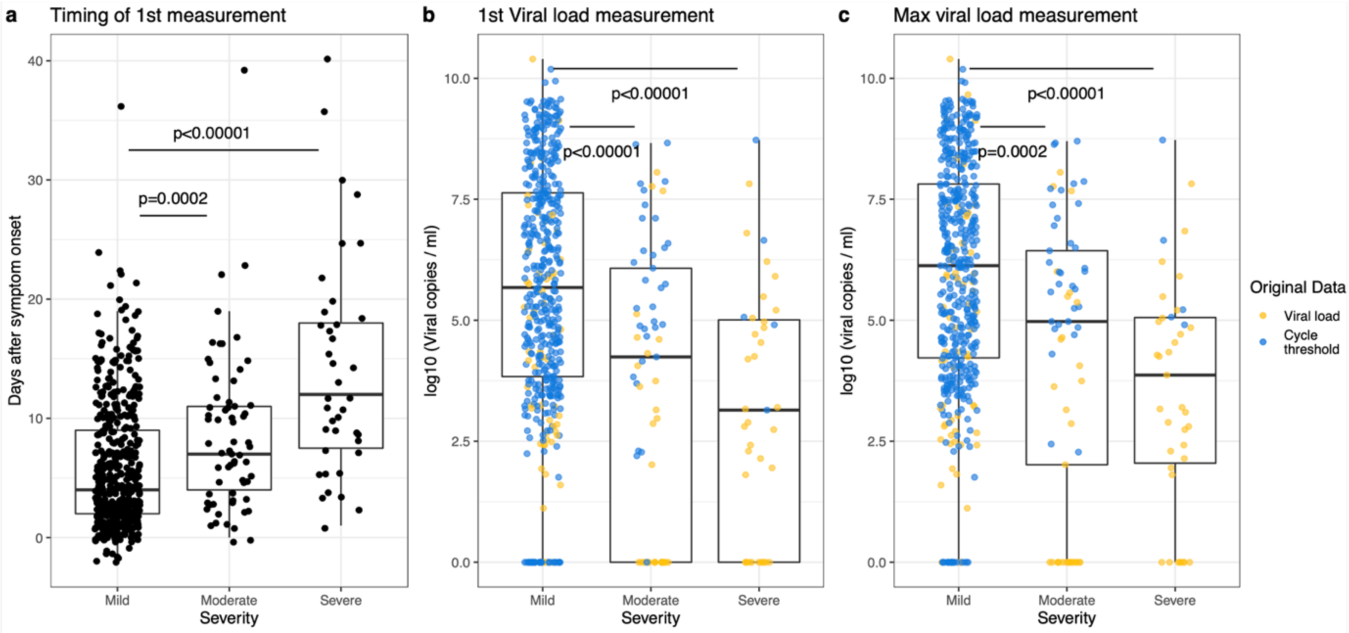

Supplementary Figure 3: Comparison of timing of first sample and viral load by severity. a) Timing of first viral load measurement for each patient, relative to symptom onset. Here severity indicates the maximum severity recorded for each patient, rather than the severity recorded at admission. We find that, on average, the first viral load measurement for patients with mild disease was recorded earlier than those for either patients with moderate disease, or severe disease (p-values from a two-sided Wilcoxon test). b) Quantification of the first viral load measurement for each patient, stratified by severity classification. c) Quantification of the maximum viral load. As shown in the left-hand panel, we have many more data points for mild patients early after the onset of symptoms, which is why both the first and maximum recorded viral loads are higher on average for this group. Across these studies, 421 patients had more than one sample recorded: for 60.1% of these patients, the first sample had the highest recorded viral load.

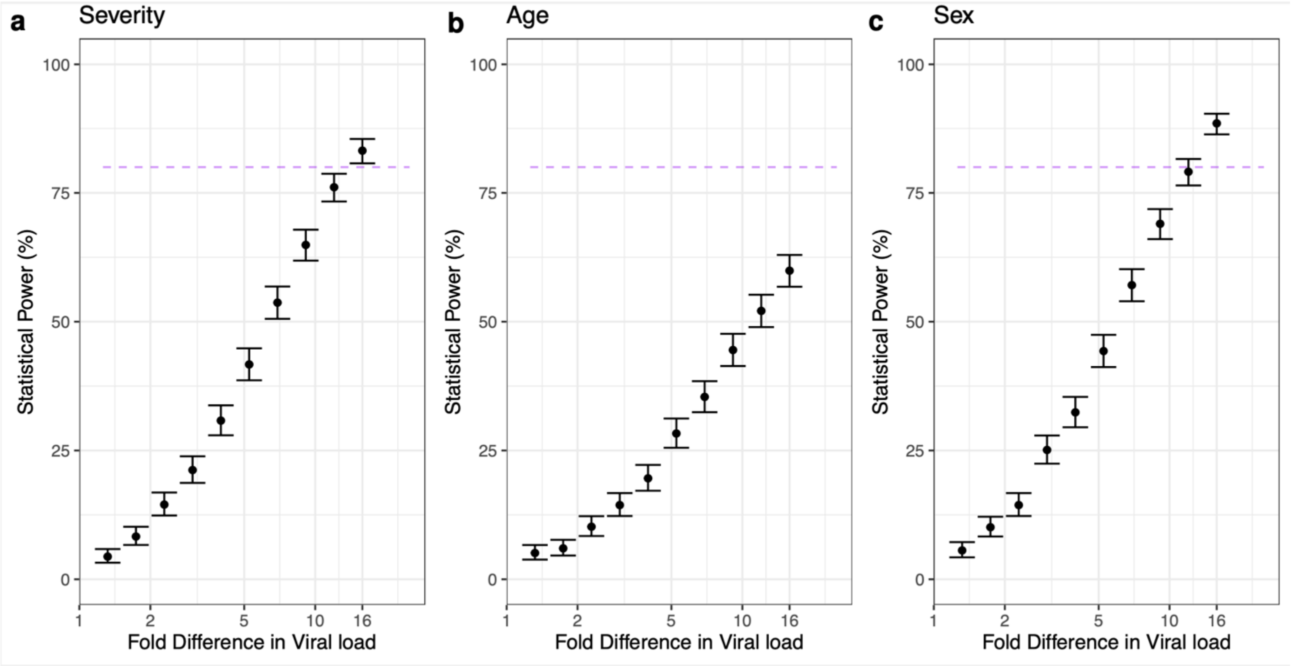

Supplementary Figure 4: Estimations of the statistical power in the regression analyses. We used simulation-based methods to estimate our power to measure an effect on the peak viral load due to severity of disease, age, or sex. We simulated datasets of the same size as the one used here for the regression analysis, containing the same level of variation between subjects and studies (Supplementary Table 3). In each set of simulations, the peak viral load was influenced by one of these 3 variables (panel (a): severity of disease; panel (b): age; panel (c): sex). In each case 1000 simulations were generated and the statistical power (black dots) calculated as the proportion of cases for which a significant effect (a fixed effect with a p value < 0.05 was deemed to show significance). The error bars show the 95% confidence intervals for each proportion calculated. In each panel, the purple, dashed line indicates 80% power. These plots suggest that we were underpowered to detect relatively small differences in viral load due to age, sex, or disease severity.

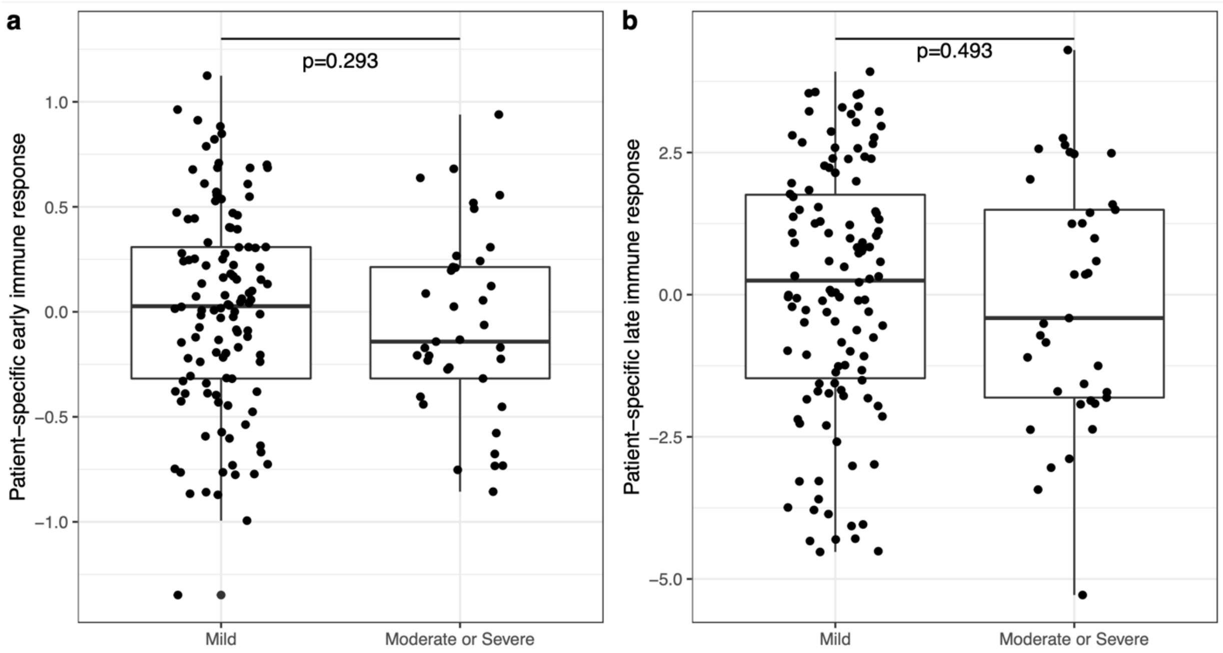

Supplementary Figure 5: Relationship between patient-specific parameters governing the immune response in the mechanistic model and disease severity. After adjusting for study (through the study-specific random effect) no significant differences were found between either the patient-specific early (panel a) or late (panel b) immune response parameters in the severity groups. This is consistent with the findings from the regression modelling. In this plot we show the posterior mean value of the subject-specific parameters.

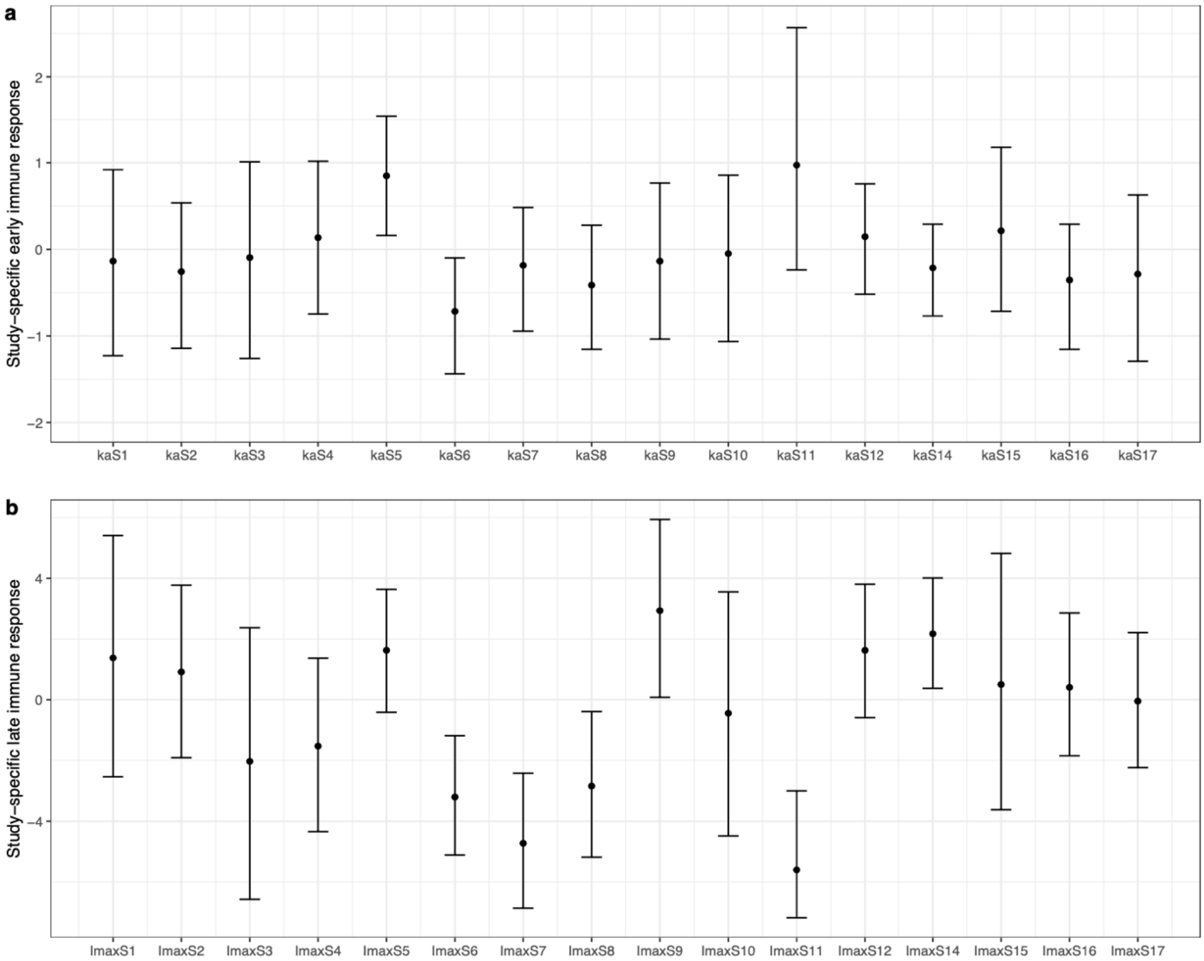

Supplementary Figure 6: Posterior means and 95% credible intervals for the study-specific offsets in the mechanistic model. Panel (a): the parameter values for the early immune response, which influences the peak viral load. Panel (b): the parameter values for the late immune response, which governs the rate at which the viral load is cleared.

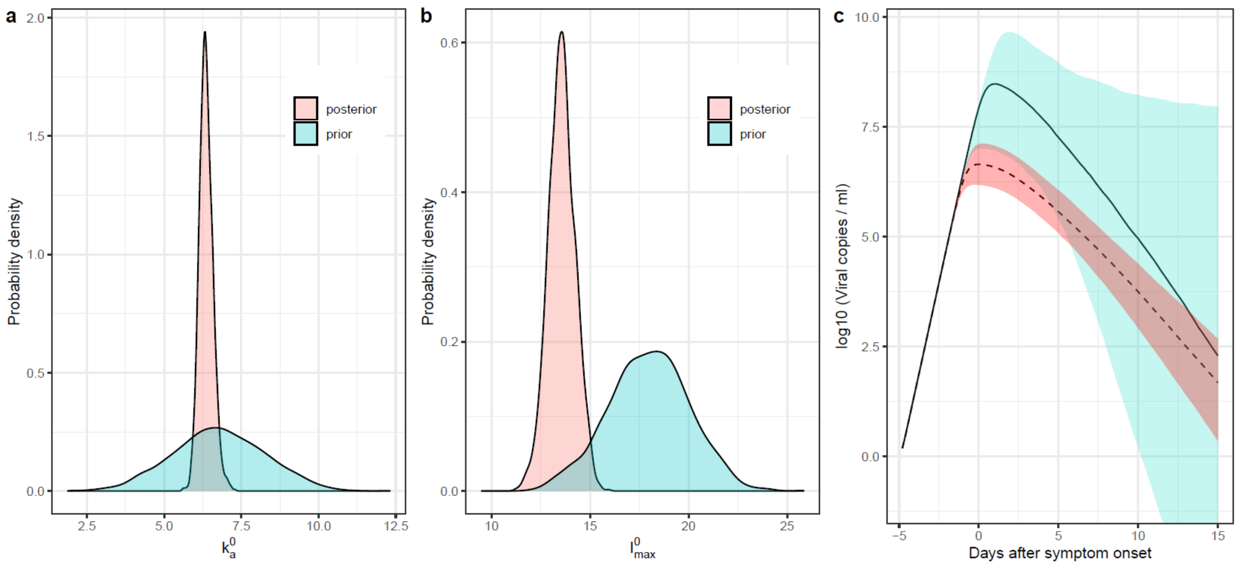

Supplementary Figure 7: A comparison of the prior and posterior distributions for the early and late immune responses in the mechanistic model. Panel (a): here we contrast the prior distribution for parameter k_a^0, which governs the strength of the late immune response (see Equations 6 and 7 in the main text). The prior distribution is shown in blue, and the posterior distribution is shown in red. Panel (b) shows the prior and posterior distributions for I_{max}^0, which governs the density of infected cells required to activate the early immune response. In panel (c) we show the dynamics generated by the mechanistic model when samples from prior (blue) and posterior (red) distributions are used for the early and late immune responses. The dynamics generated from the posterior distribution are the same as those shown by the black line and dark grey shaded region in Figure 5.

Supplementary Figure 8 *(shown over the following 7 pages)*: Output from the mechanistic model alongside the data, for all 155 patients considered. In the heading of each panel, the first number indicates the study (studies numbered as in Table 1). The second number identifies the patient. The coloured points indicate the data: for illustrative purposes, samples that were negative for virus are set to 1 viral copy per ml. The black lines indicate the model fit for each patient (calculated from the posterior mean). The shaded area shows the 95% credible intervals for the model fit.
